# Comparative Study of Alcoholic Extracts of Different Syrian Grapevine and Olive Leaf Cultivars for Their Antioxidant Activity and Photoprotective Effects

**DOI:** 10.1155/2024/7027281

**Published:** 2024-09-20

**Authors:** Farah Alhakim, Antoun Laham, Jameela Hasian

**Affiliations:** Department of Pharmaceutics Faculty of Pharmacy Damascus University, Damascus, Syria

## Abstract

A safer alternative made of plant extracts is needed, as evidenced by the negative effects of using synthetic sunscreen. Antioxidant properties of plants with high phenolic content have been reported. The goal of this research was to ascertain the phenolic content and antioxidant characteristics of ethanolic extracts made from grape and olive leaves under various extraction settings. The extracts were subjected to both qualitative and quantitative analyses using HPLC. Soxhlet extraction with 80% ethanol (v/v) as the solvent produced a result that was satisfactory. Four components of grape leaf extract and one component of olive leaf extract were identified. Expressed as gallic acid equivalents (GAEs), the total phenolic content (TPC) of the samples, as determined by using the Folin–Ciocalteu's reagent, ranged from 38.39 to 72.78 mg/g dry extract of olive and 65.918–132.7 mg/g dry extract of grape. An ethanolic extract of Salmoni (grape leaves) had the highest TPC (132 mg GAE/g), while an ethanolic extract of Zaity (olive leaves) had the lowest (72.66 ± 0.46 GAE/g). The Folin–Ciocalteu method proved the existence of antioxidants in the plant. By scavenging free radicals such as DPPH (2,2-diphenyl-1-picrylhydrazyl), the antioxidant capacity of the plant extracts was determined. Next, the ethanolic extracts of various cultivars of grape and olive leaves were analyzed to determine their sun protection factor (SPF) value. It was 28.8 and 29.96 for grape leaf extract and olive leaf extract, respectively.

## 1. Introduction

About 40% of sunlight is visible light, 10% is ultraviolet radiation, and 50% is infrared radiation. We are only interested in ultraviolet radiation (UV) because of its harmful effects on the biological organism. UV-A (315–400 nm), UV-B (280–315 nm), and UV-C (200–280 nm) are the three categories of ultraviolet radiation [1]. UV-A and UV-B radiation, however, can reach the surface of the earth and harm the skin. Changes in collagen and elastic fibers are among the skin's partially harmful effects. The associated photocarcinogenic injuries lead directly to DNA damage which increases the risk of skin cancer and accelerated skin aging. Using sunscreen to protect the skin from the sun can help reduce these risks [2, 3].

Through their ability to absorb UV radiation from the sun, the chemicals in sunscreen can protect the skin. Sunscreen's ingredients have the potential to protect human skin from UV radiation and prevent a number of skin conditions [4]. Chemical or organic sunscreens and mineral-based or inorganic sunscreens are the two groups of sunscreens that are distinguished by their mechanism of action [5].

Unfortunately, some worries remain regarding the safety of UV filters, since the negative effects of using sunscreen can include allergic and irritant contact dermatitis, photosensitivity, and phototoxicity. Concerns have also been raised about the possible hazards associated with the buildup of UV filters in the environment. Due to this, research on using natural ingredients to lessen skin irritation and other negative effects of sunscreens is continually growing [6, 7].

The naturally occurring chemical substances known as phytochemicals are biologically active substances that fall under a number of different headings, including terpenes, alkaloids, flavonoids, phenols, and saponins. Potent phytochemicals from plants that may be used therapeutically have drawn more attention from researchers in recent decades [8]. This is due to the fact that a large number of phytochemicals have been demonstrated to possess antioxidant properties that either prevent or slow down substrate oxidation and can, in some cases, even more effectively than endogenous antioxidants, delicately mitigate the effects of reactive oxygen species (ROS) [9, 10]. In the human body system, polyphenols are antioxidants that can be crucial in the adsorption and neutralization of free radicals, the quenching of singlet and triplet oxygen, or the breakdown of peroxides that can harm proteins, lipids, DNA, and other biomolecules [11–13]. In addition, cosmetics containing herbal ingredients are better suited for hyperallergic skin since they do not cause comedogenic effects, are easier to adjust to the skin, and cause less irritation [14, 15]. So, they are harmless and inert having multiple biological actions and are also protective against various diseases [16].

Thus, phenolic compounds such as flavonoids help in protecting the skin from excessive exposure to UV rays, particularly in preventing free radical damage to the skin by blocking UV rays [3, 11, 17], this proves that they have antioxidant and UV-protective properties [18].

Medicinal plants are regarded as a local treasure of global significance [19], with between 50,000 and 75,000 plants being utilized globally in traditional and modern medicine alone [20]. The use of plants in traditional diets, customs, and traditions has been documented by botanists, folk medicine, as well as folk cuisine, in different parts of the world [21]. Information from the literature on *Vitis vinifera* L. (grape) and olive indicated that the leaves of plants are a source of phenolic compounds [22, 23]. Due to their hemostatic and astringent properties, grape leaves are used in traditional medicine to treat a variety of conditions, including hepatitis, diarrhea, vomiting, bleeding, varicose veins, hemorrhoids, inflammatory disorders, pain, stomatitis, atherosclerosis, and diseases related to free radicals [24–26]. While olive trees (*Olea europaea* L., Oleaceae) and their leaves are widely used in traditional herbal medicine to prevent and treat a wide range of illnesses, particularly in the Mediterranean region [27], they were also used in the past to treat fever and other illnesses such as malaria [28], as well as to protect the body from chronic illnesses such as diabetes and cardiovascular disease [29].

The aim of this work is to study the antioxidant properties of ethanolic extracts of grape leaves and olive leaves in addition to their total phenolic content, and then determine the sun protection factor (SPF) number of them. The collected data hope to provide some basic information that may ultimately highlight the potential of these leaves as a new source of natural antioxidant-photoprotective substances with functional properties.

## 2. Materials and Methods

Ethanol, ascorbic acid, acetonitrile, and fumaric acid (HPLC grade) were obtained from Panreac Quimica SLU, Spain; caffeic acid, gallic acid, *trans*-coumarin, and quercetin standards (HPLC grade) were purchased from Titan Biotech, India; oleuropein standard (HPLC grade) and Folin–Ciocalteu's phenol reagent were purchased from Sigma-Aldrich Company, USA, DPPH reagent was purchased from Cayman Chemical, USA.

### 2.1. Preparation of the Leaves' Extracts

Various cultivars of grape leaves, such as Helwani, Faytamoni, and Salmoni, were gathered from the rural areas of Homs, Zaidal. While we obtained leaves from different cultivars of the olive tree, including Nipali and Mousaabi from Homs and Khouderi and Zaity from Tartous in Syria which were collected directly from the trees in September 2021.

The leaves were cleaned with tap water, allowed to dry at room temperature, and then ground in a mill to a fine powder. The plant powder was kept in the dark at room temperature until it was time to extract the grounded material, which was performed in different ways as follows:

- 10 g of grape leaves was extracted using a Soxhlet apparatus with 250 mL of ethanol/water (80 : 20 v/v) for 4 h at 60°C (V ext 1) [30].
- 10 g of the sample was also extracted using 250 mL of alcoholic solvent (ethanol/water) (80/20, v/v) at 60°C for 60 min (V ext 2) [25].
- Another 10 g of grape leaves was extracted using 100 mL of distilled water for 1 h at 95°C (V ext 3) [31].
- In addition, 10 g of the sample was extracted in a beaker by adding 200 mL of distilled water and heating it to 30–40°C on a hot plate while stirring continuously for 20 min (V ext 4) [32].

As for olive leaves, the extraction was performed as follows:

- 10 g of the olive leaf sample was extracted using a Soxhlet apparatus with 250 mL of ethanol/water (80 : 20 v/v) for 4 h at 60°C. (O ext 1) [30].
- 10 g of the sample was extracted with ethanol/water (80 : 20) in a water bath at 60°C for 1 h (O ext 2) [25].
- 10 g of the sample was extracted with hot water (250 mL) for 30 min (O ext 3) [23].

After that, the extracts were filtered and allowed to cool to room temperature, and then the samples were placed in a rotary evaporator under a vacuum for two hours at 50°C. The concentrated extracts were refrigerated between 2°C and 8°C [32] until HPLC analysis was performed to determine the gallic acid, caffeic acid, trans-coumarin, and quercetin contents in grape leaves and the oleuropein content in olive leaves. Next, various cultivars of grape and olive leaves were treated to the optimal extraction technique based on HPLC results, and the same compound's content was examined again using HPLC in the same settings.

### 2.2. Determination of Polyphenolic Compounds in Grape and Olive Leaf Extracts by HPLC

Using a HPLC system with a silica-based C18 bonded phase column (C18, 250 mm × 4.6 mm ID, 5 *μ*m) from Agilent Technologies, the identification and quantification of gallic acid, caffeic acid, *trans*-coumarin, and quercetin from the extracts (V ext 1, 2, 3, 4) were carried out [33]. The mobile phase consisted of A (aqueous formic acid, 98 : 2 v/v) and B (acetonitrile acid, 98 : 2 v/v) at a flow rate of 1.0 mL/min at 30°C. The detection was monitored at 360 nm for quercetin and 280 nm for gallic acid, caffeic acid, and trans-coumarin. For both the standard and sample solutions, 5 *μ*l is the injection volume that is used.

The identification and quantification of oleuropein from the extracts (O ext 1,2,3) were carried out using a high-performance liquid chromatography (HPLC) system with a silica-based C18 bonded phase column (C18, 250 mm × 4.6 mm ID, 5 *μ*m) from Agilent Technologies. The mobile phase consisted of a mixture of water and acetonitrile (80/20 volume ratio) with 1% acetic acid, flowing at a rate of 1.0 mL/min. Oleuropein determination was performed using a UV detector at 240 nm, with an injection volume of 20.0 *μ*l for both standard and sample solutions [34].

Based on retention times relative to standards, compounds found in extracts of grape and olive leaves were identified. Peak area and calibration curves from the standard solutions were used to calculate their concentrations in the extracts.

### 2.3. Analysis Extracts by Infrared (IR) Spectroscopy

All investigations on the grape and olive leaf extract samples were performed with an IR Shimadzu. The mid-infrared region (500–4000 cm^−1^) is the wavenumber range that has been set.

### 2.4. Determination of the Total Phenolic Content

The Folin–Ciocalteu reagent method was utilized to ascertain the total phenolic content (TPC) in olive leaf extract. 1 mL of the diluted extract was mixed with 4.8 mL of distilled water, 4 mL of Na2CO3 (2% w/v), and 200 *μ*L of the Folin–Ciocalteu reagent. The mixture was then left to stand at room temperature for 60 minutes. At 760 nm, absorbance was measured [35].

With only minor adjustments, the Folin–Ciocalteu method was used to determine the TPC in grape leaf extracts. 1 mL of each diluted extract was combined with 800 *μ*L of the reagent, and the mixture was left to react for 8 min before 800 *μ*L of sodium carbonate solution (0.075 mL^−1^) was added. The mixture was left to stand at room temperature (20 ± 3°C) for one hour in the dark. A spectrophotometer was used to measure absorbance at 760 nm [36].

### 2.5. Scavenging of DPPH Radical

The method outlined in the previous study [37] was used to track the DPPH radical scavenging effect of phenolic compounds found in olive and grape leaf extracts. A solution of 0.1 mL was mixed with 120 *μ*M of DPPH in ethanol. After shaking the mixture vigorously, the decrease in absorbance was measured at 517 nm until the reaction reached a steady state. Ascorbic acid, a synthetic reference and stable antioxidant, was utilized and absolute ethanol was used as the control. The following equation was used to determine the DPPH radical scavenging activity as a percentage of the sample:

(1) $$\begin{array}{c} \text{DPPH scavenging activity}\left(100\%\right)=\left(\frac{1-A517\;sample}{A517\;DPPH\;solution}\right)*100. \end{array}$$

The ratio of the tested compounds' concentration to their antiradical activity percentage was plotted to determine the IC50 values using the linear regression method.

### 2.6. Determination of SPF

1 mg/L and 50, 100, 150, 300, and 500 *μ*g/mL of the ethanolic extract were the final concentrations achieved by dissolving it in ethanol. The SPF model used in this study was according to the methodology described by Mansur et al. [38]. Using ethanol as a blank, triplicate measurements of the sample absorbances were made at each point in the UVB wavelength range (290–320 nm) in 5-nm increments. By applying the Mansur equation, the SPF was determined as

(2) $$\begin{array}{c} \text{SPF spectrophotometric}=\text{CF}*\underset{290320}{\sum }\text{EE}\left(\lambda \right)*I\left(\lambda \right)*\text{Abs}\left(\lambda \right), \end{array}$$

where EE is the erythemal effect spectrum, *I* is the solar intensity spectrum, Abs is the absorbance of the sunscreen product, and CF is the correction factor (=10) [39, 40].

The values of EE × I are constants and are shown in Table 1.

### 2.7. Statistical Analysis

Three samples of olive and grape leaf extracts of each treatment were independently analyzed in each sampling, and all of the determinations were carried out in triplicate. The results are expressed as means ± standard deviations. All statistical analyses were performed using the Windows Excel 2020 program.

## 3. Results and Discussion

### 3.1. Determination of Polyphenolic Compounds in Grape and Olive Leaf Extracts by HPLC

Following extraction, the extract is dried until it reaches a steady weight. By comparing the chromatograms of the standards, the identification and quantification of oleuropein from the olive leaf sample and gallic acid, caffeic acid, *trans*-coumarin, and quercetin from the analyzed grape leaf sample were accomplished. Using a Soxhlet apparatus and 80% ethanol as a solvent produced the best results when compared to other extraction methods, as evidenced by HPLC chromatograms that displayed a high concentration of compounds in the extract.

During the first phase of research, the chemical composition and raw material quality were assessed in order to choose a variety with the best biochemical composition for a reliable source of sun products. In order to ascertain the concentration of gallic acid, caffeic acid, *trans-*coumarin, and quercetin in the grape leaf samples and oleuropein in the olive leaf samples, the next step involved analyzing three different cultivars of grape leaves and four different cultivars of olive leaves in Syria. By comparing the compounds' chromatograms with standards obtained under particular experimental conditions, the compounds were identified.

**Figure 1:**
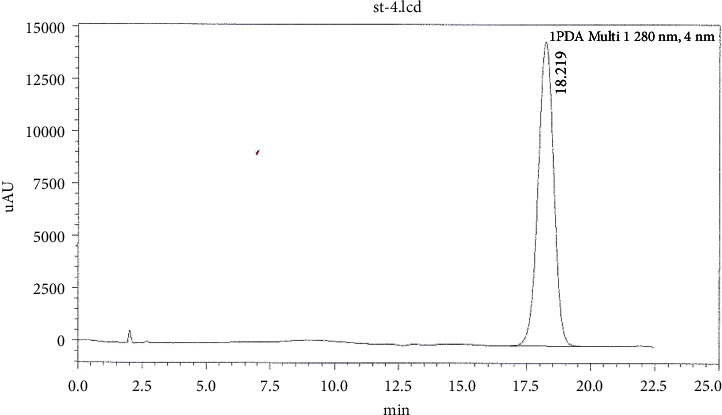
HPLC chromatogram of oleuropein standard.

**Figure 2:**
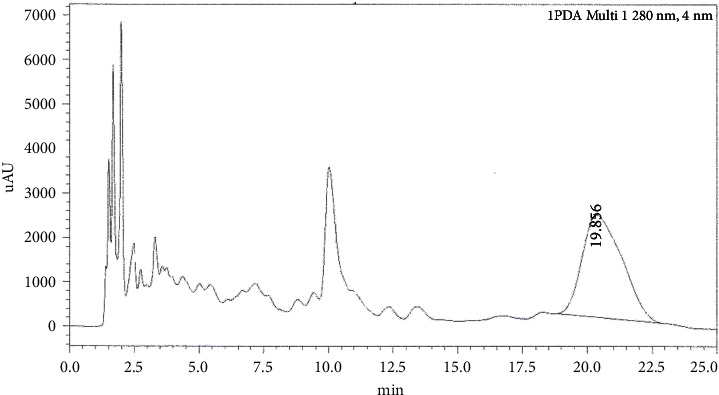
HPLC chromatograph of oleuropein for olive leaf extract (Mousaabi).

**Figure 3:**
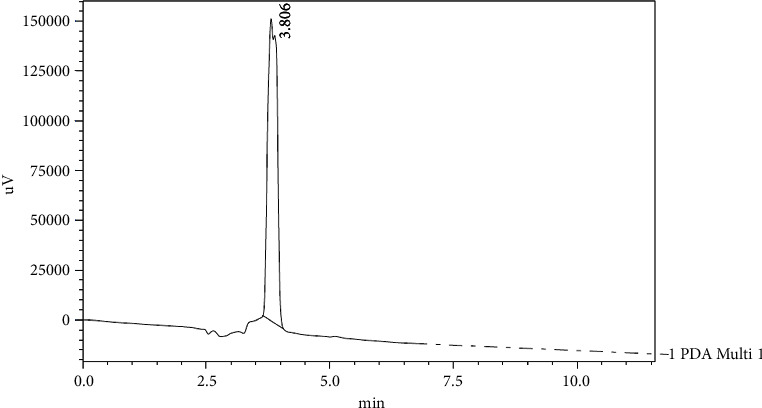
HPLC chromatograph of gallic acid standard.

**Figure 4:**
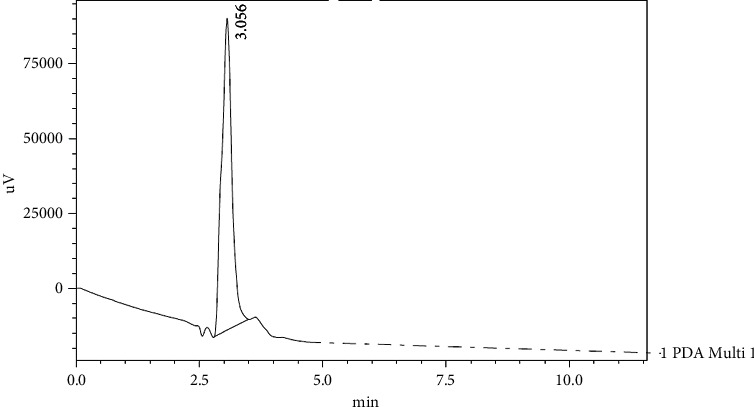
HPLC chromatograph of caffeic acid standard.

**Figure 5:**
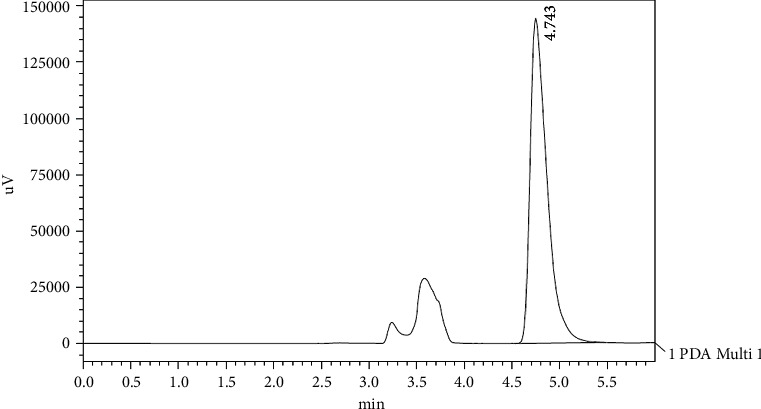
HPLC chromatograph of *trans*-coumarin standard.

**Figure 6:**
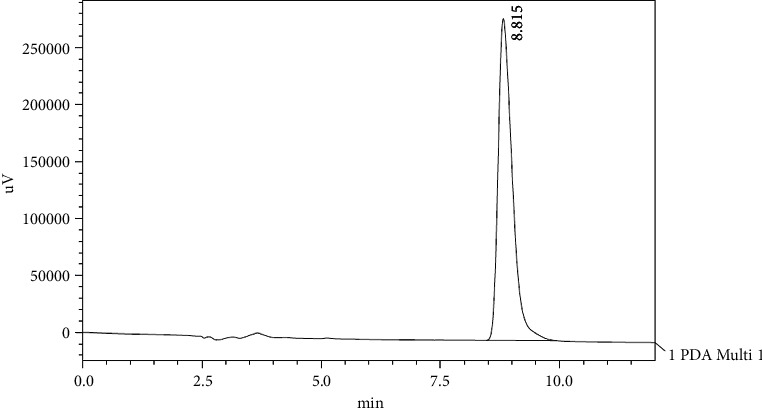
HPLC chromatograph of quercetin standard.

Figure 1 shows a chromatogram of a standard solution of oleuropein with a retention time of approximately 18.219 min.  Figure 2 shows the chromatogram of oleuropein in Mousaabi olive leaves which gave the best result among other varieties. Figures 3, 4, 5, and 6 show chromatograms of a standard solution of phenolic compounds for the determination of the grape leaf extract.

**Figure 7:**
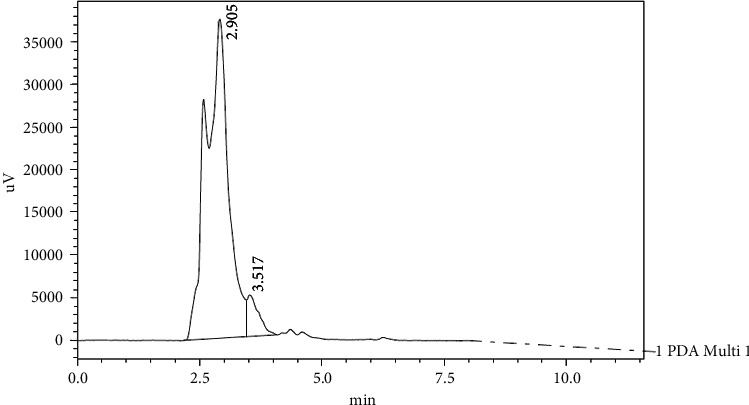
HPLC chromatograph of gallic acid and caffeic acid for grape leaf extract (Helwani).

**Figure 8:**
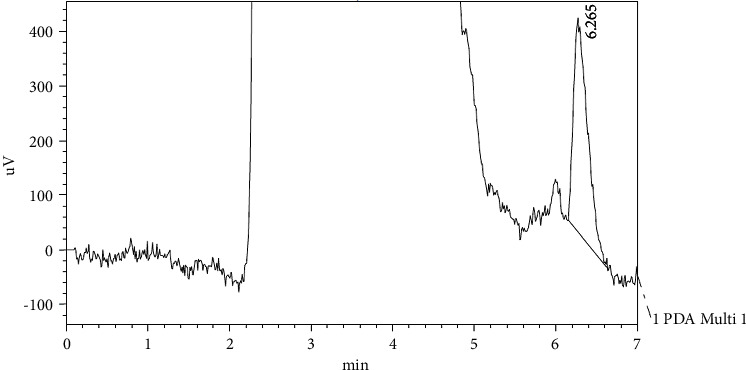
HPLC chromatograph of *trans*-coumarin for grape leaf extract (Helwani).

**Figure 9:**
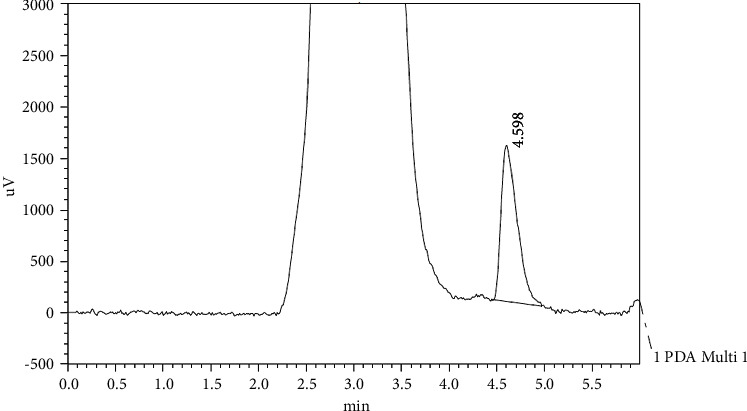
HPLC chromatograph of quercetin for grape leaf extract (Helwani).

Figures 7, 8, and 9 show the chromatograms of the abovementioned compounds in samples of Helwani grape leaves which gave the best result among other varieties.

Tables 2 and 3 present the mean values of gallic acid, caffeic acid, *trans-*coumarin, and quercetin in the grape leaf extracts of the three cultivars, and oleuropein in the olive leaf extracts of the four cultivars.

In this study, the oleuropein concentration results ranged between 1.63 and 7% for different varieties. According to references [27, 41], the approximate value of oleuropein concentration was about 1–14%. All experimental data of olive leaf extracts were compared with an oleuropein standard in order to obtain a reliable characterization.

However, using the Soxhlet apparatus for extraction of grape leaves revealed higher concentrations of quercetin and gallic acid than those found in reference data [22, 24].

### 3.2. Analysis Extracts by Infrared (IR) Spectroscopy

**Figure 10:**
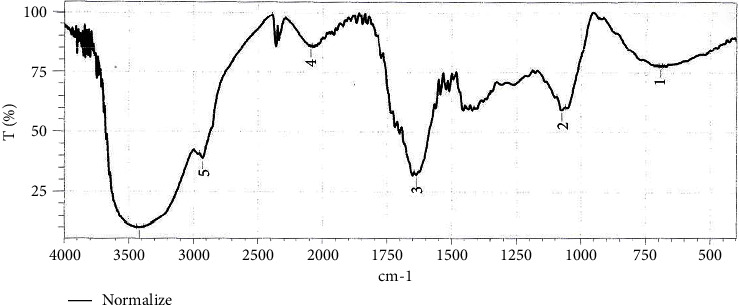
IR spectra of grape leaf extract.

**Figure 11:**
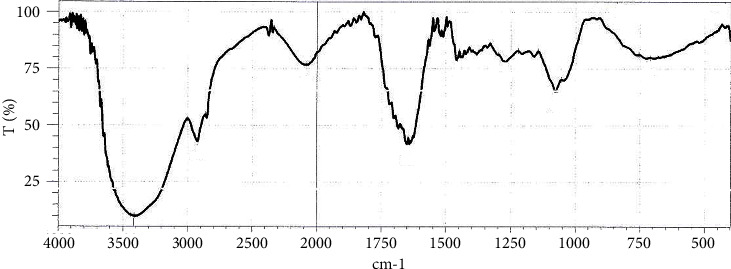
IR spectra of olive leaf extract.

Absorption bands were observed in regions around 3500 cm^−1^ with wide shoulder shapes representing O-H (phenolic and hydrogen bonding), in the region around 3000 cm^−1^ for C-H aliphatic stretch, in the region around 1620 cm^−1^ for double band C-O stretch, band around 1100 cm^−1^ corresponding to C-O phenols, and the band in the region between 650 and 950 cm^−1^ for aromatic C-H bending (Figures 10 and 11) [42] The IR spectra emphasizes structure of some compounds found in grape leaf and olive leaf.

### 3.3. Antioxidant Activities

The present investigation has demonstrated that while grape and olive leaves are rich in phenolic compounds, the concentrations of these compounds vary amongst the cultivars.

A calibration curve was created using multiple concentrations of gallic acid standards in order to obtain a linear equation for the assay, which used a calibration curve equation, in order to determine TPC. Equation *y* = 0.0013*x* + 0.2954 yields the calibration curve, and the correlation coefficient (*r*) is equal to 0.999 [43–45].

The TPC results for the olive leaf extract in Table 4 indicated that the extracts of various cultivars ranged from 38.39 to 72.78 mgGAE/g, with the extract from Mousaabi having the highest concentration at 72.78 mgGAE/g and the Salmoni extract had the highest concentration, with the TPC of grape leaf extract ranging from 65.918 to 132.7 mgGAE/g (Table 4).

Comparable results to ours, as reported in [46], ranged from 54.23 to 97.58 GAE/g in various soil compositions, with the same TPC result for various grape leaf cultivars (Helwani and Faytamoni). However, when compared to the same study, the Salmoni leaf extract yielded the highest result, although comparable values, ranging from 77.3 to 179 mg GAE/DW, were reported by Kocsis [47] for a number of Hungarian cultivars.

Our findings on olive leaves are generally consistent with those of Turkish olive cultivars documented in the literature [28].

The DPPH assay technique relies on the stable free radical DPPH being reduced. Maximum absorption of the single-electron free radical DPPH occurs at 517 nm (purple color). Antioxidants interact with DPPH, a stable free radical that conjugates in the presence of a hydrogen donor (e.g., a free radical scavenging antioxidant), and is reduced to DPPHH and as a result, the absorbance from the DPPH. In comparison to the DPPH-H state, this radical version causes decolorization (a yellow hue) as the number of electrons collected increases. The more color removal the more the reducing ability [48].

Faytamoni (grape leaves) and Mousaabi (olive leaves) were found to have higher DPPH activity, whereas Helwani (grapevine leaves) and Nipali (olive leaves) had lower DPPH activity. Comparing this study with another on Syrian olive leaves, it was observed that the results were higher for TPC and similar for IC_50_ [35].

Thus, the high total phenolic content in grape and olive leaves may suggest that they have potent antioxidant properties. In two tests, grape cultivars, particularly Faytamoni and Salmoni, exhibited the highest levels of antioxidant activity. As there is a direct correlation between phytochemical content and antioxidant activity, high phenolic content in extracts is actually typically a good indicator of antioxidant properties [49].

### 3.4. SPF Value Determination

The ability of natural products to shield against UV radiation damage has been assessed by recent studies. Plant extracts have active components that can scavenge free radicals, prevent enzymatic degradation of the skin matrix to protect it, or stimulate the synthesis of collagen to improve the elasticity and hyperpigmentation of the skin. Furthermore, a variety of phytochemicals, including flavonoids and phenolic compounds, have aromatic structures that can rapidly return to the ground state after efficiently absorbing photons, much like UV filters. These structures are occasionally linked to carbon-carbon double bonds and/or carbonyl moieties [50]. Recent studies have concentrated on the use of phenols and flavonoids, which absorb UV light, as antioxidants in sunscreens to offer photoprotection. The application of natural antioxidants in the prevention of UV-mediated diseases is now possible as a result of this [39].

SPF values, which indicate the amount of time one can spend in the sun without getting sunburned, have evolved into a universal metric for evaluating the efficacy of sunscreen products. Concentration had an effect on the SPF values of the tested extracts; an increase in concentration led to an increase in SPF. This action is explained by the phenolic compound present in extracts of olive and grape leaves.

After calculating the SPF, the absorbance of the solutions was measured between 290 and 320 nm, with a 5 nm measurement range, which corresponds to the UVB radiation wavelength. Among the other cultivars, Helwani and Mousaabi had the highest values (28.8–29.9) for 1 mg/mL, respectively. Different grape and olive cultivars contain phenolic compounds that may be responsible for this activity.  Table 5 displays the sun protection factor (SPF) value for various cultivars of grape leaf and olive leaf extract.

## 4. Conclusion

Taking protective measures is essential to prevent sunburn, photoaging, and skin diseases caused by excessive exposure to sunlight, which promotes the generation of ROS and RNS, thus leading to oxidative stress.

SPF is a quantitative measurement of the effectiveness of any sunscreen formulation, and it is important to protect the skin from excessive sun exposure, which can lead to oxidative stress, sunburn, photoaging, and skin diseases. Although many synthetic sunscreens are available, their use is limited due to their harmful effects on the skin. Consumers are more accepting when sunscreens are formulated with plant extracts. Apart from filtering UV radiation, herbal formulations have many beneficial effects on the skin.

There is an increasing interest in plant polyphenols due to their antioxidant, anticancer, and many other benefits for human health.

In this study, olive and grape leaves were tested for their total phenolic content and antioxidant capacity. In summary, it has shown a very good potential antioxidant property.

The presence of phenolic compounds in the leaves was confirmed by the Folin–Ciocalteu method. The antioxidant capacity was measured by DPPH free radical scavenging methods and was found to be high.

The SPF values of different Syrian cultivars of ethanolic grape and olive leaf extracts were evaluated. Most of them have the ability to protect against UV radiation. Besides their many beneficial effects and safety, these plants could become a good, cheap, and easily available formulation ingredient in sunscreen products.

## Figures and Tables

**Table 1:** Values of EE × I.

Wavelength (nm)	EE × I (normalized)
290	0.0150
295	0.0817
300	0.2874
305	0.3278
310	0.1864
315	0.0839
320	0.0180

**Table 2:** Contents of phenolic compounds in grape leaf extracts.

Cultivars	Quercetin (*μ*g/g)	*Trans*-coumarin (*μ*g/g)	Caffeic acid (mg/g)	Gallic acid (mg/g)
Helwani	56.25	3.75	0.19	3.75
Faytamoni	41.25	1.88	0.37	5.63
Salmoni	31.25	2.06	0.31	4.38

**Table 3:** Contents of oleuropein compound in olive leaf extracts.

Cultivars	Oleuropein (%)
Khouderi	3.5
Mousaabi	7
Zaity	1.63
Nipali	2.87

**Table 4:** Determination of total phenolic contents (GAE, g1) and free radical (DPPH) % scavenging of grape leaf cultivars.

Cultivars	TPC mg/g extract	IC_50_
Olive leaves		
Mousaabi	72.78 ± 0.767	0.13 ± 0.002
Khouderi	38.39 ± 0.127	0.61 ± 0.017
Zaity	59.84 ± 0.774	0.45 ± 0.102
Nipali	59.16 ± 0.679	0.86 ± 0.008
Grape leaves		
Salmoni	132.74 ± 0.1739	0.4 ± 0.014
Helwani	65.97 ± 0.177	0.82 ± 0.009
Faytamoni	71.374 ± 0.146	0.12 ± 0.001

**Table 5:** SPF value calculation of ethanolic grape and olive extracts for different cultivars.

Cultivars	50 *μ*g/ml	100 *μ*g/ml	150 *μ*g/ml	300 *μ*g/ml	500 *μ*g/ml	1 mg/ml
Grape leaf						
Faytamoni	0.92	3.37	5.52	9.29	14.49	25.45
Helwani	1.77	3.80	5.06	10.26	18.04	28.80
Salmoni	1.45	1.68	3.21	6.17	12.78	22.76
Olive leaf						
Zaity	0.95	1.22	3.16	4.29	4.29	14.48
Khouderi	0.71	1.95	4.60	12.43	16.57	19.65
Nipali	2.65	7.21	10.54	20.95	27.17	27.91
Mousaabi	0.67	0.88	2.20	5.37	8.90	29.96

## Data Availability

The data used and/or analyzed during the current study are available from the corresponding author on reasonable request.
